# Stakeholder analysis in urban-planetary health research: The Key Group Approach

**DOI:** 10.3389/fpubh.2025.1629249

**Published:** 2025-09-24

**Authors:** Daniel Black, Geoff Bates

**Affiliations:** ^1^Population Health Sciences, University of Bristol, Bristol, United Kingdom; ^2^Daniel Black + Associates | db+a, Bristol, United Kingdom; ^3^Institute for Policy Research, University of Bath, Bath, United Kingdom

**Keywords:** stakeholder analysis, stakeholder identification, complex research, planetary health, urban development

## Abstract

**Introduction:**

There is growing recognition of the importance of ensuring that research involves stakeholders who both affect and are affected by the problems under investigation. However, this presents significant challenges for researchers seeking to solve global problems such as disease prevention and planetary health, and for many reasons: e.g. scale and complexity of systems, high number and inaccessibility of stakeholders, and the range of understandings of what ‘the problem’ is. Methods are needed to help research teams ensure that those recruited are as representative as possible.

**Methods:**

This approach was developed as part of a programme of research in the United Kingdom that sought to improve decision-making in order to prevent diseases linked to unhealthy urban environments including those linked to climate change. The work evolved over the 8 years of research, but was prompted ultimately by the final year of the programme in order to improve the quality of the programme-level stakeholder evaluation workshops. The method was developed by integrating a narrative review of the literature with foundational programme theory and emergent theory of change in order to develop key principles, criteria and conceptual understandings. This led to the development of 16 core stakeholder typologies, a comprehensive database structure and simplified partner-focused checklist, and 14 points for discussion. These were refined through the stakeholder identification and recruitment process into the final approach presented here, which includes a retrospective gap analysis.

**Findings:**

The final approach and toolkit includes a step-by-step process over three rounds of iterative and integrated research activity, combined with supporting checklists, principles, categories and questions. Teams seeking to involve stakeholders in urban development or similar planetary health research can use these to interrogate their samples in order to understand both representativeness and alignment to programme theory and mission. No context is the same, so each approach needs to be tailored to suit. We describe common principles, and an example of how the toolkit was applied in our research study. We reflect on the process using the points for discussion identified, and demonstrate how analysing our sample in this way helped us to understand and identify both strengths and limitations.

## 1 Introduction

### 1.1 Planetary health and the challenge of stakeholder involvement

The aim of this article is to provide a method for stakeholder analysis intended for use when researching complex societal problems, including those relating to planetary health. Specifically, it can support the identification of representative stakeholders, or the analysis of samples of stakeholders to understand how representative they were. This challenge of ‘stakeholder analysis’ (including both the identification of stakeholders and analysis of those identified) has been investigated by various disciplines over the years, such as business ethics and healthcare, but most especially perhaps by those concerned with long-term sustainability, systems thinking and co-production [e.g., (1–8)]. The emerging view from the UK public health research community is that “*new approaches to population health research are clearly needed*” and we suggest - in the context of planetary health research especially - that must include our (collective) approach to stakeholder analysis (UKPRP, 2018). It appears to be particularly important within planetary health research for a number of reasons, including: the scale and complexity of system, the very wide range and high number of stakeholders, the need for real world solutions, combined with considerable uncertainty and lack of shared understandings of the problems (9–11). However, there remains a lack of guidance to support the design and delivery of transdisciplinary collaborations and the involvement of stakeholders in sustainability and planetary health research (12).

The method proposed here is designed to support those who aim to maximise stakeholder involvement, working within inevitable resource and time constraints, in order that there is a shared understanding of the systems being investigated that is as well-rounded and nuanced as possible. Given the sectoral focus, it should be of particular and immediate use to anyone working at the interface between urban development and public or planetary health, whether in research or practice, and specifically when considering how to involve stakeholders in a range of data collection activities, including workshops, focus groups, interviews or surveys. It should also be adaptable to almost any context that requires stakeholder analysis and identification, and be especially useful when dealing with complex real-world problems with very significant numbers of linked stakeholder groups.

The introduction continues with a narrative literature review charting some of the main methodological developments in stakeholder analysis and identification over the last 30 years. Building on the key concepts from this review, in the third part of the introduction we then set out how we have developed our own approach for stakeholder analysis in research, which aimed to enable the creation of healthy urban environments in the United Kingdom (UK). In section 2, we describe a replicable method of stakeholder analysis based on our own approach. In the results (section 3) we demonstrate how applying this method in our research enabled the identification both of a representative sample of stakeholders to support the evaluation of our research programme, as well as the clear identification of gaps to support critical reflection and any further ongoing engagement. In section four, we discuss the advantages and limitations of this method, issues of replicability and working with hidden stakeholder attributes, and suggested improvements.

### 1.2 Developments in stakeholder analysis and identification

Stakeholder analysis and identification is not a new area of interest in academic research (13–15), but it appears to be generating interest more recently within the world of transdisciplinary research [e.g., (16, 17)], and more specifically in research aimed at solving complex global challenges, because of the large number of stakeholders and the associated challenges of identification and involvement [e.g., (16, 18)]. We consider here some of the key concepts and ideas for stakeholder analysis as they have developed over the past 35 years.

Significant attention was given to this area across disciplines spanning philosophy and business ethics, renewable energy, sociology and international development. The focus then primarily appeared to be on making clear the difference between those stakeholders who are affected by an issue and those who have influence over it. Goodpaster (1991, p.56), for example, a philosopher interested in business ethics and corporate responsibility, describes stakeholder analysis as a process of perception and analysis such that “*the affected parties caught up in each available option are identified and the positive and negative impacts on each stakeholder are determined*.” He also sets out four further steps in a sequence – *synthesis*, *choice*, *action* and *learning* - with synthesis separate from analysis, and set out as *“structured information according to whatever fundamental priorities obtain in the mindset of the decision-maker*.” In other words, his use of the term analysis is the process that stakeholders go through to analyse information, rather than the identification of those stakeholders. Nonetheless, his work recognises the distinction between those affected by and those affecting an issue, and their respective interests and motivations, concepts that are developed by others in the coming decades. Babiuch and Farhar (19), sociologists working on renewable energy, describe stakeholder analysis as a process that allows analysts to identify how parties are likely to be affected by government projects and programmes, which involves identifying the likely impacts of a proposed action and the affected stakeholder groups. Notably, they do not appear so interested in identifying those making the decisions, which may reflect their focus on major energy infrastructure (and the relatively more limited number of those decision-makers in that context). Schmeer (15), a sociologist working on health reform in international development, described stakeholder analysis as “*a process of systematically gathering and analysing qualitative information to determine whose interests should be taken into account when implementing a policy or programme*.” More specifically, she sets out a section on stakeholder identification, recommending that “*the working group should identify all actors who could have an interest in the selected policy”* including actors outside the health sector that could affect or be affected by the policy and naming specific sectors and organisations of relevance (15, p. 8).

In the late 2000s and 2010s, in Canada, the UK and Australia, there is evidence of further interest from the worlds of anthropology, environmental management and population health respectively, with some conceptual development moving methodological approaches into the domain of systems theory. Chevalier and Buckles (20), social anthropologists who specialised in participatory action research, make more explicit the disaggregation of those ‘affecting’ from those ‘affected’ in two ways: firstly, by presenting a middle ground where stakeholders may both affect and be affected by the issue, and secondly, grading the extent to which each are affected by splitting each across three levels: least, moderately, and most. Reed (18, p. 1937), an academic focused on environmental management, extends this area further by bringing in notions of ‘problem understanding’ or ‘problem identification’, making the case that a “*clear understanding of the issue under investigation*” is needed, with “*the boundaries of the phenomenon clearly defined”* and *“based on well-founded criteria established by the research analyst.”* Reed sets out six stages of stakeholder identification: (i) identify focus e.g., issue, organisation or intervention; (ii) identify system boundaries; (iii) identify stakeholders and their stake; (iv) differentiate between and categorise stakeholders; (v) investigate relationships between stakeholders; (vi) recommend future activities and stakeholder engagement. [It’s worth noting that this approach continues to be followed in Norway by Guise et al. (3), albeit in the healthcare and resilience setting]. Bammer (16), a population health scientist, echoes both Reed in terms of the critical need for understanding the problem area, and Chevalier and Buckles by suggesting that stakeholders are “*all those groups who have a practical grasp of the problem* (p. 16)” and that it can be useful to think about them as: a) those affected by the problem, and b) those in a position to influence the problem. In 2016, Reed subsequently developed stakeholder analysis templates to encourage researchers to think more methodically about stakeholders’ level of (i) ‘interest’ in the research area and their (ii) ‘influence’ on its outcomes. Through this period, therefore, we appear to see a development from thinking about who is involved and to what extent, to a recognition of the need to be clear about the exact nature of the problem(s) under investigation, and whether all involved have a clear shared understanding of that ‘problem space’.

In the 2020s, there is evidence from the UK, Philippines, the USA and notably Switzerland of increasing interest and sophistication in how this area is approached. Balane et al. (21), public health researchers, focus on issues of power in the world of global health policy. They suggest a number of specific challenge areas - fast-changing policy environments, number of stakeholders, ability to delineate personal versus role-driven opinions, sensitivities around power and interest, and potential bias of analysts – and develop a finalised framework of four main areas: (i) *knowledge*; (ii) *interest*; (iii) *power*; (iv) *position*. Pohl (17), an environmental scientist - contributes with a method called ‘actor constellation mapping’, though the initial stakeholder identification and recruitment appears later via his colleague (22), a professor of science communication, who adds a method called ‘constellation analysis’, which seeks to map relations between four types of elements: social actors, natural elements, technical elements and signs/symbols, a process within which identification (of social actors) is a first step. Around this same time, Bammer (23) draws on a range of literature in her Stakeholder Engagement Primer (10), including Mitchell et al. (7), which suggests four criteria for selecting stakeholders – (i) legitimacy; (ii) real and potential power; (iii) the urgency they assign to the problem; (iv) practical considerations – and the use of table/checklists and mind-maps to help with the identification, each of which have detailed meanings. Legitimacy refers to: value of their experience and insight, whose interests they serve, how representative, and level of participation. Power refers to issues that are: utilitarian (e.g., access to data, funding, etc.), normative (e.g., prestige, esteem), and/or coercive (e.g., force, vested interests). Urgency refers to: perceived importance and time sensitivity. Practicalities refers to: access to stakeholders, relationships, willingness to participate, realistic in terms of what is achievable, and risks. Mitchell and colleagues (24) revisit this work to review what has changed in the 20 years since they started working on stakeholder analysis and identification in the corporate and business sectors, and specifically on the salience framework of power, legitimacy and urgency. They conclude that, despite enormous progress in stakeholder theory and research, “*managers may or may not perceive who their stakeholders are and whether/how they are important or salient* (p. 871),” suggesting there is still some way to go.

**Figure 1:**
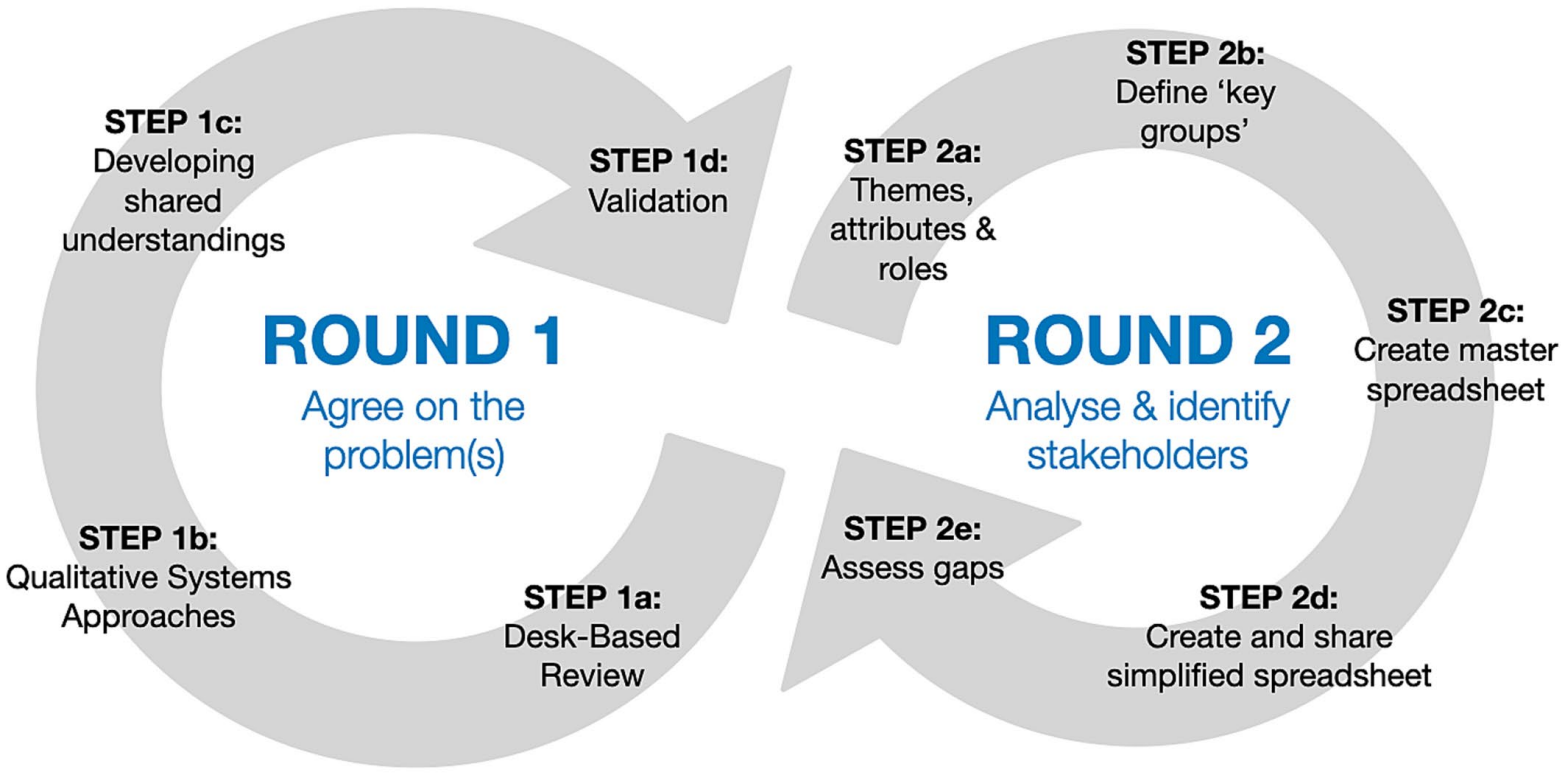
Stakeholder analysis and identification over two rounds, repeated as and when possible, starting with a main focus on agreeing the problem(s).

In reviewing these conceptual and methodological developments, we can infer a number of core elements (Figure 1). At the most superficial level, the most obvious requirement is that stakeholders should include both those affecting – those with influence over an issue - as well as those affected by an issue. In addition, however, the extent to which stakeholders are involved needs to be known, and could be categorised as high, medium or low. Under this, considerable nuance of understanding is needed, with assessment based on: knowledge; interest; power (real and potential); position; legitimacy; and their sense of urgency. Finally, there are issues of practicality that need to be taken into account.

### 1.3 The development our approach

The methodological challenge therefore is to identify and ensure - with as much confidence as reasonably possible - that those involved in the research are as representative as possible of all relevant stakeholders, and will have collective knowledge that is as comprehensive as possible about the problem space (16, 18). Lack of representation and time to fully deliberate and include all those who should be included is a challenge even in more ‘traditional’ stakeholder involvement [e.g., (25, 26)]. In planetary health research, these issues can become exponentially challenging due to the scale and complexity of the systems under investigation. It is critical therefore to assess carefully who needs to be involved, bearing in mind the need for adjustment as new evidence emerges (27). For the same reason, stakeholder analysis is not a one-off activity, but will likely need to be repeated many times during research for different purpose of involvement (e.g., defining the scope of research, acting as project advisors, participating in the research or engaging with findings and evidence). We argue that to undertake rigorous stakeholder analysis for these purposes, research teams need to have (i) a shared understanding about the problem space being investigated in order to identify who relevant stakeholders are, (ii) clarity on the areas of knowledge and experience that are needed to provide a comprehensive understanding of this problem space, and (iii) nuanced understanding about who the key stakeholder groups are in this system.

We demonstrate the stakeholder analysis method through its application for the evaluation of a six-year research programme, ‘Tackling Root causes Upstream of unhealthy Urban Development’ (TRUUD), which investigated UK property development and transport planning systems in the UK, and then implemented multi-action interventions to prioritise health in these decision-making processes (28). The TRUUD research team included academics in six UK universities who led the research activities, working with a range of external partners in the public and private sectors, including public representatives from the two city regions being used as case studies, all of whom provided insight and guidance throughout. The programme evaluation aimed to understand the cumulative effects from intervening in multiple and cross-sectoral areas of decision-making. We sought to engage stakeholders in a series of workshops to interrogate the emerging evidence from TRUUD, help us to understand future impacts, and identify barriers and facilitators. Our challenge was to bring together stakeholders with a collective breadth and depth of knowledge and understanding about this complex system of systems, and to ensure this sample was representative of the range of stakeholders affecting and affected by the interventions implemented in the research.

Our research group developed its shared understanding of the problem space - including clarity of the knowledge areas needed across our stakeholder sample and awareness about different stakeholder groups - primarily through the data gathering undertaken during both main phases of the programme of research (2019–2024) as well as the preceding pilot study (29). Each of the activities, summarised in Table 1, contributed to the development of our understanding of key groups of stakeholders and how they were related to the problem space. We do not anticipate that all research studies will undertake all these activities, not least given inevitable limits in time and resource across varying research projects and programmes. However, it should help to demonstrate how understanding develops over time, and also that stakeholder analysis undertaken at the start of research might differ from that at the end, by which time more nuanced or advanced understandings of the problem space and stakeholders will have been developed.

**Table 1:** Activities undertaken that informed the development of our stakeholder analysis approach.

Stage	Activity
Pilot study (29)	Early conceptualisation and development of two foundational understandings (31, 53): 1. The lack of clarity on what is meant by the term ‘upstream’ (i.e., in the public health world, this can mean anything upstream of ‘downstream’ health outcomes) and the suggested need therefore for use of new term: ‘midstream’ 2. Recognition of the critical importance of power and influence, and the need for clearer understanding of this in order for engagement to be effective, especially with influential decision-makers
	Early development of stakeholder templates setting out specific attributes drawing on Reed’s (30) -e.g. *interest*, *influence*, *motivation*, *interests*, *needs* – and adding our own: *time horizons*, *local interest*, *capacity and resource issues.*
	Key themes identified in the interview analysis (31): *prioritisation of health, economic valuation, short-termism, partnership, land, public realm, role of Government, reconciling tensions.*
Protocol (28)	Key areas for consideration developed as part of foundational programme theory with new and wider research team (and later revisiting and revising of this) covering: *role of fast growing cities, land ownership, development delivery process, finance, complexity, innovation in public involvement.*
Main research phase 1 (33)	Categorisation of stakeholder influence and interest in interview sampling, leading to internal debates and concerns about how to assess this meaningfully.
	Development of sub-categories for private sector, responding to concern around lack of nuance and contextual awareness (see Table 2).
	Systems/boundary mapping focusing on actors across the system (54), Figure 4 working across three dimensions: *context, stakeholders, themes/concepts.*
	Numerous themes were identified during much expanded interview and workshop analysis, but final analysis led to three core themes: *agenda setting, structural barriers, existing points of leverage.*
	Identification of areas of intervention for development in the research programme (see Table 2)

In going through these activities, we developed a checklist of prompts with which to analyse (and identify) each stakeholder, and to ensure that the final sample of stakeholders was representative of the key groups needed. The checklist used ideas of stakeholder interest and influence (30) as a starting point, with developments over time based upon the findings of the research activities. For example, through the analysis of pilot interviews we identified 10 headline themes of systemic barriers preventing healthy urban development: agenda-setting and prioritisation; balanced, comprehensive valuation; short-termism and corporate governance; balanced partnership; land control; land value; identifying ‘good’ partners; the public realm challenge; the role of government; reconciling tensions (31). Through the analysis of a far larger set of interviews in TRUUD we gained a more nuanced understanding of these themes, and added new ones (32). These interviews and analysis helped us to understand the complex urban development decision-making space, and the range of stakeholders involved and their expertise. In addition to building on our empirical data, revisiting and revising our programme theory was also essential due to the complexity of the systems under investigation (27). In our protocol (28) we highlighted the roles of: valuation mechanisms; fast growing cities; the dominance of private sector actors (landowners, developers, investors); complex systems of governance; and the need for meaningful public engagement. These led to additional prompts in the checklist, alongside a final set representing seven areas of decision-making that the research ultimately identified to directly intervene in (‘intervention areas’): national government funding and appraisals, corporate behaviour change, real estate investment, city-region transport planning, spatial planning for large-scale development, law and planning, and public involvement (33).

Specific exercises of stakeholder analysis undertaken as part of these research activities also contributed to the final checklist and, specifically, the categories of stakeholders that needed to be included. For example, we drew on Reed (30) initially again in the first phase of the main research project when seeking to identify who to recruit for interviews. For this we compared potential participants on ‘interest’ and ‘influence’ ranked as high, medium and low. Stakeholders were also categorized more straight-forwardly according to ‘scale’ (international, national and local), ‘sector’ (public, private, third, individual) and ‘focus’ (e.g., urban planning, transport, property developer, land/real-estate, housing, health, finance/investment, environment, construction, community). Due to the wide-ranging complexity of the private sector, those targeting this sector had to undertake a further group categorisation, listing as exhaustively as possible all the various sub-sectors (e.g., real estate investors, developers, pension funds, insurance, house-builders, financial services, consultants, brokers, agents, banks, landowners, technical specialists) with rationales next to each in order to sort into an assessment of ‘essential’ and ‘non-essential’.

By putting all these criteria and prompts together with the findings of the literature review, we identified 24 questions relating to areas of knowledge needed, and two sets of categories relating to types of stakeholder roles and their experience relating to the problem space (e.g., power, influence, interest; Table 2). These then formed the basis of the stakeholder analysis for our programme evaluation workshops. In our case, the analysis was undertaken collaboratively by a small sub-team within the research consortium working with public sector partner organisations based within the three cities in the UK that the workshops were delivered in: Cardiff, Edinburgh, and Birmingham. Using the method provided in the next section we firstly sought to set out the categories of stakeholders to include in the evaluation. Secondly, for each location we identified multiple individuals in each of these categories and used this to, thirdly, identify the final sample to participate in the workshops.

**Table 2:** Initial stakeholder analysis framework.

	Do we have stakeholders who understand…	Categories 1 (from stakeholder categorisation)	Categories 2 (from literature review)
Pilot - themes	How to prioritise health in decision-making? The need for economic valuation (and how to apply it)? How to overcome short-term thinking? What balanced partnerships might look like? Issues of land control and value? How to address the issue of maintaining the public realm? The role of government in this challenge space? How to reconcile the tensions identified?	Scale: international, national, local Sector: public, private, third Focus/disciplines: Urban planning, Transport, Property development, Land/Real-estate, Housing, Health, Finance/Investment, Environment, Construction, Community Essential private sector sub-sectors: Pension Funds/Insurance (Institutional Property Investors); Real Estate Investors (Other); Volume Housebuilders; Developers (Commercial / Residential / Mixed-Use / Independent); Financial services and real estate agencies; Brokers - Real Estate; Brokers - Land agents; Consultants – Surveyors; Consultants - Accountants/ Management consultants; Consultants - Property Lawyers; Consultants – transport; Consultants - Planning/Design/Engineering Public: statistically, politically, geographically or experientially representative	Knowledge Interest Power (real and potential) Position Legitimacy Urgency Practical considerations Additional questions: What influence does the stakeholder have over the problem? What are their motivations? What are their needs? What time horizons do they work to? What is their local interest? (see notes) What capacity and resource issues should we bear in mind? What weaknesses might there be in their involvement?
Main study	How agendas are set (and how health can be prioritised)? Global and structural barriers and how they might be overcome? How to coordinate existing points of leverage?		
Intervention areas	National government? Corporate behaviour change? Real estate investment? City-region transport planning? Spatial planning for large-scale development? Law and planning? Public involvement?		
Programme theory	The role of (fast growing) cities? Landownership (in relation to development)? Development delivery processes? Investment (in relation to development)? Complex systems of governance? Public involvement that transcends current practice?		

## 2 Materials and methods

**Figure 2:**
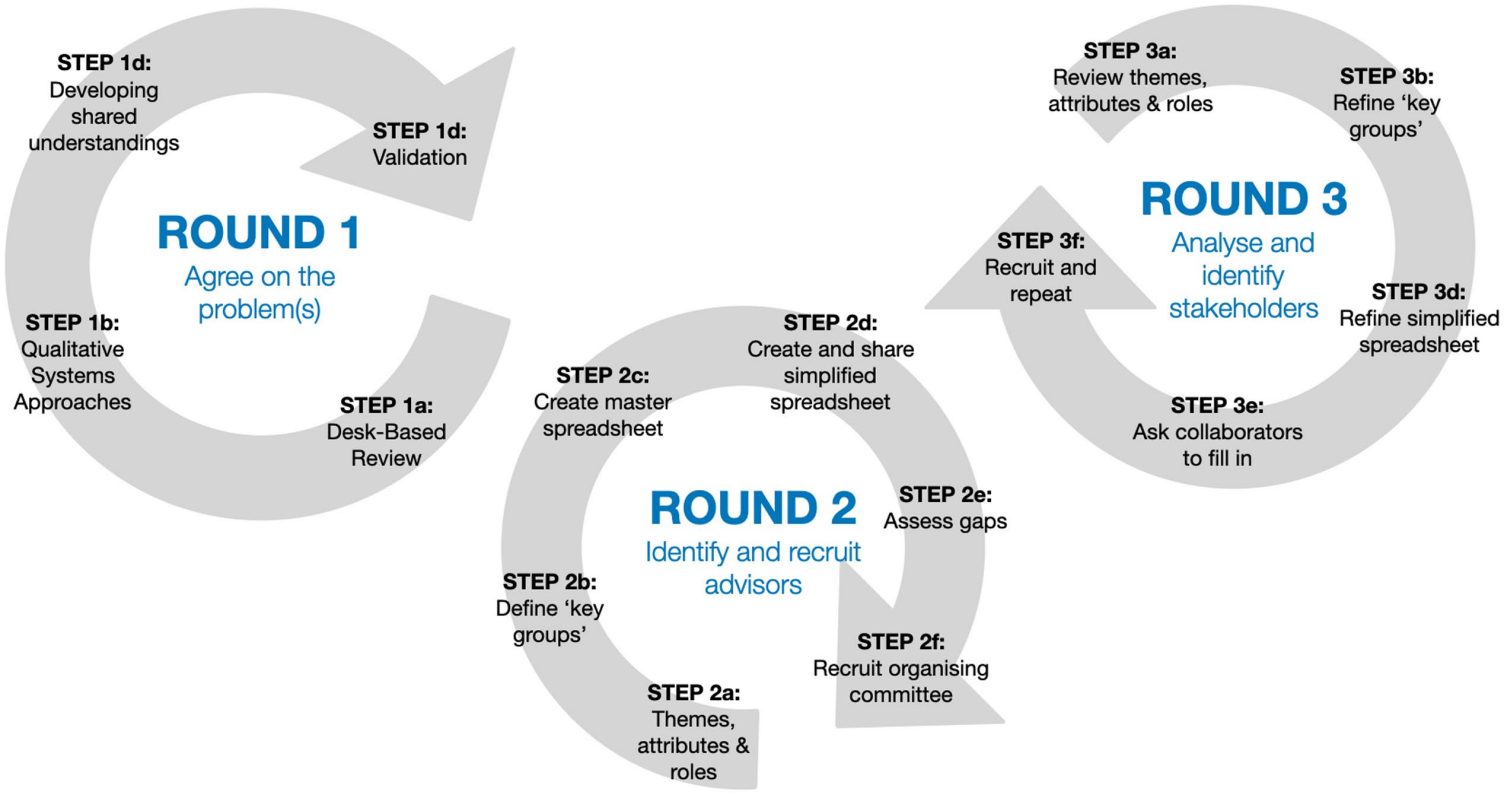
Stakeholder analysis and identification augmented with additional middle round to identify advisors.

In this section we provide a replicable method that can be used to identify and ensure - with as much confidence as reasonably possible - that the people involved in research are as representative as possible of all relevant stakeholders, and collectively have a breadth and depth of understanding about the system(s) under investigation. It enables the identification of gaps and, as such, can also be used in retrospective analysis of stakeholders to understand how well this has been achieved. As set out above, it’s essential to revisit and revise iteratively the research group’s shared understandings about the problem and who should be involved in the research (27). As such, each of the main steps of the approach can cycle back to any other stage (Figure 2), but broadly they include:

1. Agreeing of the main (root cause) problem(s)
2. Identifying key themes
3. Defining the ‘key groups’
4. Creating shareable template(s)
5. Assigning ‘key groups’
6. Assessing gaps
7. Filling gaps where possible

Problem identification is such a considerable exercise in itself however, that we split these steps into two different, albeit linked rounds (Figure 1). We agree with Reed (18), that this first step of problem identification stands slightly apart from the main process of stakeholder analysis and identification. Unlike Reed, however, we would suggest it is important to undertake, regardless of the perceived understanding of the problem, given the complexity of planetary health challenges and inherent uncertainties as set out above. It is important to continue developing shared understandings of the problem in order to identify stakeholders to involve, and why. We suggest it might help to adopt principles used in ‘root cause analysis’, which prompt researchers to look for the underlying causes, albeit bearing in mind the principle within systems theory that there are no root causes as all elements interact with each other (34).

### 2.1 Round 1: Agreeing on the problem(s)

We suggest the following five steps, and associated questions, are needed in agreeing on the problem space to enable effective stakeholder analysis. We anticipate that often these steps will be part of an existing programme of a work, rather than an additional set of tasks, and that agreements of the problem to be addressed through the research will also be informed by other factors such as programme theory and funder requirements.

#### 2.1.1 Step 1a–Desk-based review

Firstly, what does the literature say about the problem (and about the problems that cause the problem)? Ideally, this can be co-produced with as wide a range of people as possible (within reason), to combine theoretical understandings and set out your group’s starting position [e.g., (28)].

#### 2.1.2 Step 1b - Qualitative systems approaches

Secondly, what do other people say is the problem, or are the main problems, and specifically the problems that cause those problems? In planetary health research, this can seem overwhelming due to the complex nature of these challenges. There is an inevitable initial challenge in determining the boundaries of the systems under investigation. Ensuring the research team combines broad contextual understanding and expertise both in systems approaches alongside qualitative methodological rationale is therefore important. For example, when considering sample size, as set out by Vasileiou et al. (35) there can be many justifications, most commonly these are ideas based on saturation and pragmatism, but all can be debated and this becomes essential given inevitable resource constraints, even on very large programmes. They conclude by urging both transparency in reporting on project needs (and constraints) and a stronger focus instead on ‘*data adequacy’* or *‘evidentiary adequacy’* (as opposed to saturation). This expands the concept beyond having a sufficient number of participants to include their variety, relevance and credibility. This, in turn, is reliant on strong foundational theory grounded in understanding of systems approaches (16, 36, 37).

#### 2.1.3 Step 1c – Developing shared understandings

Thirdly, how might you develop accepted shared understandings based on the findings from steps 1a and 1b? There are many different ways of doing this such as through group discussions and workshop activities to come to some agreement about the nature of the problem, what needs to change and where in the system, and what types of interventions might be needed. Having clear ‘shared mental models’ that represent shared beliefs about the system can be useful [e.g., Hall et al. (54)]. Such models can be developed through a wide range of approaches (38), not just group model building (39) or systems mapping activities [e.g. (40, 41),], though these latter approaches offer a particular level of granularity if used well. While not essential on all projects, it can be very useful to be supported by those who specialise in such techniques.

#### 2.1.4 Step 1d: Validation

Finally, how can you validate the findings? This will already have been achieved to greater or lesser extent by the steps above, especially if a relatively comprehensive involvement of stakeholders is achieved through those steps, but additional validation can be sought where feasible from any external advisors to check understandings and to inform the direction of the research. The familiar challenges remain in terms of representation and understanding of who will ideally be involved, and hence where possible all steps should be revisited after the steps below have helped map the relevant actors (and associated system boundaries) [(18, 42), Figure 4].

As understandings of the problem space develops, it is worth bearing in mind an often-overlooked factor in the development of shared understandings, especially on large research projects, which relates to effective internal communication (43). It is likely that many of the activities above have been undertaken by different individuals and groups in the research team, all of whom are starting with different understandings of the problem. These understandings are complex and built up over time, so even after considerable efforts, within the relatively short timescale of a research project understandings are likely to remain quite different. Investing time and resource into high quality internal communication, and providing clarity of mission, management and leadership (43, 44), are therefore important to manage expectations and, within reason, reduce uncertainty and tensions in the team (45–47). However, plural understandings are inevitable and even desirable (48, 49).

### 2.2 Round 2: Analysing and identifying stakeholders

Having spent time making the problem space as clear and as explicit as possible, the next step is to turn the resultant understandings into a practical rationale for stakeholder identification and analysis, which can be used by the research team. This round involves the creation of a series of tables, each building on the previous, to create a practical checklist of categories that can be used to ensure that relevant attributes and expertise are covered in a representative sample of stakeholders during their recruitment, or to be applied retrospectively when analysing the sample. A key innovation, we argue, is the project-specific description and application of ‘Key Groups’ (Step 2b onwards), which provide a ‘short-hand’ for describing those who cover critical knowledge domains, thereby enabling rapid assessment across the research team (and external partners if collaborating).

#### 2.2.1 Step 2a - Develop attributes and identify specific roles

We recommend starting by turning the themes identified through the development of your understanding of the problem space into questions on the areas of expertise needed, starting with: ‘Do we have stakeholders who understand…?’ as demonstrated in Tables 2, 3 (Table 3 gives two examples from a much larger table, which is provided in the Supplementary material). Describe as richly as possible the rationale underpinning each question/theme, then list the different sectors (e.g., private, public, third, academia) and example roles from each that you would expect to hold these attributes (e.g., developer CEOs, social impact leads, chief planners).

**Table 3:** Shortened version of table used to develop key groups from themes, attributes and example roles.

Do we have stakeholders who understand…				
	Specific attributes	Example roles	‘Key Group’ title	Description
How to prioritise health in decision-making?	Clear understanding of key influential decision-making and impacts on health outcomes. Most experienced urban practitioners are aware of what’s healthy, but very few have a comprehensive understanding of what is needed.	Private: CEOs, Experienced Developers/Investors with track record in high quality development, Public: Senior officers from relevant departments at national and local level, Chief Planner, Treasury Lead responsible for Regen/Development, One Public Estate Lead Third: Senior/experienced think tank policy specialists	Quality deliverers	Experienced urban practitioners aware of what’s healthy and what’s not (e.g. high quality homes, low car, quality green space, etc.), AND of what is needed to deliver high quality development. These tend to be developers and/or developer-investors, or social impact/ESG leads working independently or with investors, delivering higher-end, premium quality ‘products’, though even these tend to focus in high value locations where they can charge a premium.
How to overcome short-term thinking?	Notiriously challenging: requires entirely new ways of governing so likely will need experience, systems thinking and innovation (and/or indigenous wisdom?)	As above, but with innovative thinkers (e.g. RSA? Indigenous voice?)	Cathedral thinking innovators	Overcoming short-termism is a ‘wicked problem’: it requires entirely new ways of governing so likely will need experience, systems thinking and innovation. ‘Cathedral thinking’ is a concept that refers to those that built the cathedrals in the middle ages, which took 100 s of years to complete, knowing they would never see the end result.

#### 2.2.2 Step 2b - Create and define ‘key groups’

Having described each theme or area in detail, setting out their specific attributes and roles (Step 2a), assign to each row a short ‘Key Group’ name. Use positive terminology that sums up as succinctly as possible each role/area (e.g., ‘public health expert’, ‘transport strategist’) based on the themes, attributes and example roles - these terms will be different depending on your unique context and focal area(s) - and define each in detail for sharing with those contributing to the analysis (see example in Table 3). The exact terminology of the Key Group title is less important than the understanding of the team as to which knowledge domain it covers. For example we use the term ‘Cathedral Thinking Innovator’ to refer to those who can think long-term and use that to help innovate in the present (9). Ultimately, it needs to be a practical and useful short-hand for the team, which only they can determine as each context is unique.

#### 2.2.3 Step 2c - Set out full list of categories

Using your bespoke key groups identified, create a master table with a final set of stakeholder categories. In our case the categories included: region, public sector, private sector, third sector, technical disciplines, public representatives and ‘key group’ categories identified (see Table 3; Supplementary material), but will vary according to the scope of the research and boundaries the research take place in. This is the main stakeholder checklist and forms the basis for the template to be used for analysis.

#### 2.2.4 Step 2d - Create and share simplified template

Given the relative complexity and size of the master spreadsheet, a simpler version is needed to enable those involved in the analysis to use collaboratively (Table 4 – full version in Supplementary material). This simplified form is useful for sharing within a research team, or externally if you are involving external partners in the stakeholder analysis and identification. Re-create the list of categories into a new table, splitting each main sector (public, private, third and academia) into separate sections, and under each the tier at which they operate and area they cover, with space for names, position and organisation. Add a final column titled ‘key groups’ for the team to fill in after stakeholders have been identified. Marking stakeholders with more than one Key Group is fine given that some people cover multiple knowledge domains, though if done it must then be remembered that this will mean final numbers relate to knowledge domains not individual stakeholders. Identifying specific people within your categories can draw on different approaches such as desk-based research, snowballing and consultation with partners, or building on the networks and knowledge of team members, however the key point is to identify stakeholders who can cover the sections of the template.

**Table 4:** Example sections of final, simplified template shared internally and externally.

Sector	Tier	Area	First name	Surname	Position	Organisation	Key groups*
Public	**UK-Wide**	Government Finance Financial oversight					
	**National**	Housing and Communities Energy and Net Zero Healthcare Transport Environment and Food Land					
	**Combined authorities** **(England Only)**	Spatial planning Transport Health					
	**Local**	Planning Property Major Projects Health Sustainability					
**Private**	**Investors**	Pension funds / Insurance Real Estate Investors Banks					
	**Developers**	Commercial Residential Independent Custom Build					
	**Land**	Agents Promoters					

#### 2.2.5 Step 2e - Categorise and assess gaps

Using this new, filled-in spreadsheet, carry out quick desk-based, online reviews of each suggested stakeholder, then categorise each using one or more ‘key group’ categories. Compile new table of results to identify gaps (see Results). Ask any partners to validate findings or gaps and suggest any additions as required.

## 3 Results

In this section we provide a brief overview of the outcomes from applying this method to identify and analyse a sample of stakeholders for participation in workshops designed to evaluate the research programme. Table 5 sets out the number of Key Group representatives that were identified for each workshop and in the final sample. Green indicates high representation with no need for additional recruitment; blue indicates moderate numbers of stakeholders, which could be improved, but not essential; red indicates problematically low numbers/representation. The identified stakeholders for each event were broadly similar across Key Groups: i.e. well represented in terms of planning and public health, some representation on delivery, valuation, policy and transport, but relatively very low across a wide range of other areas. We found that viewing the sample in this way helped us to identify and fill some important gaps based on our understanding of the problem space (e.g., land, law, finance). The reduced numbers in the final sample reflects that we were limited by factors such as needing to keep total numbers relatively low in each location in order to deliver an effective workshop, and the inevitable variation in availability and interest of those identified.

**Table 5:** Table showing key group numbers, both invited and final numbers who attended, at each location.

Key group (Knowledge Domain)	Cardiff		Edinburgh		Birmingham		Key group totals	
	Identified	Accepted	Identified	Accepted	Identified	Accepted	Identified	Accepted
Quality deliverers	12	1	14	4	12	4	38	9
Valuation experts	3	2	11	3	1	0	15	5
Cathedral thinking innovators	2	1	3	0	3	0	8	1
Progressive partners	1	0	7	4	0	0	8	4
Land experts	3	2	5	1	2	0	10	3
GBI innovators	1	0	1	0	2	1	4	1
Policy innovators	12	1	19	2	23	2	54	5
Behaviour change experts	3	1	0	0	0	0	3	1
Transport strategists	7	2	3	1	10	3	20	6
Progressive planners	9	5	13	3	15	6	37	14
Systems lawyers	0	0	2	1	0	0	2	1
Urban–Rural futurists	0	0	4	1	0	0	4	1
Financial innovators	1	0	6	1	5	0	12	1
Public health experts	15	6	11	5	13	8	39	19
Public representative	0	0	2	1	1	0	3	1
Inclusivity expert	1	0	0	0	0	0	1	0
Infrastructure provider	3	1	0	0	0	0	3	1
Event totals	73	22	101	27	87	23		

Green indicates high representation with no need for additional recruitment; blue indicates moderate numbers of stakeholders, which could be improved, but not essential; red indicates problematically low numbers/representation.

There were some differences in the completed templates and numbers of stakeholders identified by workshop location (73 - Cardiff, 87 – Birmingham and 101 - Edinburgh), but at each event the number of those who accepted the invitation were relatively similar (22, 23 and 27 respectively). Across all three cities, the numbers identified were relatively plentiful across a majority of the groups, with some notably very large levels of representation (delivery, policy, transport, planning, public health), and with notably low numbers in other areas: GBI (green / blue infrastructure), behaviour change, law, rural and infrastructure (the low numbers in terms of public representation and inclusivity were expected given these events were aimed at professionals). Some – notably ‘Cathedral Thinking Innovator’ and ‘Financial Innovator’ - had relatively high number of identified stakeholders (green), but relatively very low numbers in the final sample (red).

While there were clear similarities across each location, the completed templates varied noticeably in terms of numbers and coverage. Edinburgh had broad coverage, but were well represented by valuation experts, ‘progressive partners’, planners and public health experts, but had no ‘cathedral thinkers’, ‘GBI Innovators’, inclusivity experts or infrastructure providers. Birmingham had less coverage with strong representation on delivery, transport, planning and public health, but no representation across all others apart from two policy representatives. Cardiff also had a good spread like Edinburgh, and was strong in planning and public health, but had gaps in ‘progressive partners’, ‘GBI Innovators’, law, rural, finance, public and inclusivity, and low numbers in all others. Being able to see the differences in the nature of the sample at each location was very useful later when interpreting the data that was collected at the three workshops, and exploring differences.

## 4 Discussion

In this section we discuss the advantages and limitations of this Key Group approach for stakeholder analysis, and issues of replicability. We also discuss the issue of hidden attributes, an issue we identified in the development of the approach and could not include formally in the analysis. We finish by suggesting a core improvement to the approach.

### 4.1 Advantages

A clear advantage is that it enables research teams (and their partners) to understand quickly which stakeholder groups important to the research are well represented and which are not, and therefore where to target follow-up invitations towards less well-represented groups. For example, where we identified gaps in law expertise amongst our sample we were able to identify and target additional stakeholders in that space. As stated, this challenge appears well recognised in the literature and in need of attention [e.g., (18, 21)]. This exercise was particularly revealing in that it helped us appreciate the nature of public partner networks, and the ease of access in some groups versus challenge in other groups. It also helped us identify new categories that had been missing from our initial list (e.g., ‘Infrastructure Providers’, which covered not just healthcare facilities, but infrastructure more broadly and its delivery). Previously, this would not have been as easy (or even possible) due to the size and range of potential stakeholders. Crucially, it enables this to happen in a quick and straight-forward way for any researchers, external actors or agencies involved, something that is particularly important for local government in the UK currently due to lack of resource. This ease of use should be just as attractive to private and third sector partners/advisors, and it should be more accessible to the lay public too (23, 50). An additional advantage, even if not all gaps are filled, is agreeing on critical gaps in representation. This is a clear benefit when considering these complex challenge areas. In other words, even if it is not possible to have comprehensive coverage and representation, which is common, the acknowledgement of gaps can not only help with the planning of future involvement, but also help those involved to factor in those gaps of representation as best they can by, for example, discussing and considering what those representatives might say or need. An obvious example in the case of planetary health research is those with little or no voice, such as future generations and non-human ‘stakeholders’ (the natural world) (51, 52). In our case, when reviewing the findings from our evaluation we were able to consider that gap and reflect on how it might affect our conclusions, even if we were not able to include representative voices in the research.

### 4.2 Limitations

In terms of limitations, the stakeholder identification is reliant on the knowledge and networks of those involved and will therefore be limited to greater or lesser degree (21). This may not be an issue, depending on the context, but where gaps are identified, and where networks in those areas are not well developed, there is limited scope within the method as specified above for filling those gaps. That said, where resources allow, or even in the ongoing research design, this could be mitigated considerably by designing in several rounds of identification to allow for snowball identification, recruitment and agreement. Another limitation, though more difficult to say categorically, was that the process also seemed reliant on those leading the process having a relatively unusual depth and breadth of knowledge spanning all the knowledge domains in question. Specifically, it required a considerable amount of *ad hoc* analysis of knowledge domains and stakeholders in order to generate attributes, identify positions, synthesise into key groups (Steps 2a-2c) and assess gaps (Steps 2d-e). This should be possible in most research projects given that they are usually designed around the expertise of the research leads, but it is important that experienced researchers input into these processes covering as wide a range of relevant knowledge domains as possible. More widely, the time demands on all involved from undertaking a comprehensive stakeholder analysis like this should not be underestimated. While it is possible to take a more light-touch approach to applying this method, and in some cases this may be desirable depending on resources and need, it is likely that the analysis will be weakened as a result.

### 4.3 Replicability

In terms of replicability, as set out above, agreeing on the problem(s) is deceptively challenging (16, 18). To expect all research teams to be able to undertake Round 1 iteratively - literature reviews, interviews and workshops, and be well resourced on communications and with advisors - is clearly unrealistic. That said, the principles remain the same so it should be possible to follow, to greater or lesser extent, by any team at any point in their own research journey. For example, they may only have resource for a rapid literature review, one or two small workshops, alongside *ad hoc* conversations, but the process can be followed regardless, any limitations can be stated in the reporting, and identification and recruitment can build as the research work develops. The challenge of ensuring that the relevant level of expertise and resource within the research team should be possible with appropriate design and planning up front, though we anticipate that there may well be reluctance from senior researchers to engage at this level without clear communication and agreement up front as to the value of the exercise. Beyond this, there is nothing technically challenging about Round 2 so should be easily replicable by those who have a broad understanding of the problem space. As with Round 1, additional iterations can be designed in to ensure gaps are filled.

### 4.4 Working with hidden attributes

A notable distinction between best practice as described in the literature on the one hand [e.g., (7, 10, 21, 30)], and what was possible within the method on the other, was the inability to formalise a means of assessing those ‘deeper’ stakeholder attributes identified (Table 2: knowledge, interest, power (real and potential), legitimacy, urgency). Likewise, we were unable to formalise in the process the additional attributes we identified ourselves through the development of our stakeholder analysis templates: influence, motivations, needs, time horizons, local interest, capacity and resource issues, and weaknesses (e.g., conflicts of interest). This echoes a point made by one of our experienced qualitative research leads who queried how feasible it was to assess the ‘interest and influence’ columns in the template provided by Reed (30), which we drew on in our pilot and major programme. On reflection, however, much of this information will be known – to greater or lesser extent, and whether consciously or unconsciously - by those identifying the stakeholders: the better they know the stakeholder (or stakeholder group), the more of these attributes they will be aware of. As with the mitigation described in the limitations section, therefore, if done well, these deeper understandings should be addressed and revealed through additional planning, resourcing and further iterations. In other words, best practice stakeholder analysis and identification requires time and resource to undertake properly, and those undertaking it in the context of planetary health research should allow plenty of time for iterative, snow-ball identification and recruitment.

### 4.5 Improving the approach

In reflecting on this process, one obvious lesson is how much those involved influence the type of stakeholder groups based on the networks they have direct access to. In our case, we were dominated by public health experts, albeit with some good coverage across main areas such as planning, policy and transport. We did however have a number of very significant and notable gaps in our final sample, which do need to be filled if issues of planetary health are going to be addressed in any meaningful way. Indeed, this was a key benefit of the method in that it enabled us to easily identify gaps and reflect on this when analysing the evaluation data. For example, that we only had one expert each in green and blue infrastructure, law and finance means that inevitably the data we gathered from the stakeholders involved will be much more limited in these important areas. As such, ideally, for projects engaging in planetary health arenas wanting to undertake comprehensive and rigourous stakeholder analysis, we reflect that there should be an additional middle round added to the method proposed in this article – a new Round 2 – to allow for the identification and recruitment of an advisory group that is broadly representative of (and well networked in) each of the areas identified through the first iteration of the Round 1 stakeholder identification (Figure 2). This does not need to be a formalised committee with terms of reference. It could simply be an internal list of those identified, who can provide advice on stakeholder identification based on the knowledge domains identified. This should ensure a much more even coverage of key groups is achieved.

Regardless of these limitations, and whether or not there was comprehensive coverage, this ‘Key Group’ approach provides a useful and accessible way of assessing gaps in stakeholder representation, and it should be useful in a wide range of contexts. Moreover, gaining a better understanding about a sample, and how representative or otherwise it may be, is valuable for interpreting research findings.

## 5 Conclusion

Stakeholder analysis is not new, but it’s an area of research design that we suggest needs much greater attention nonetheless, especially in planetary health research where the systems under investigation are highly complex, and involve innumerable stakeholders, agencies, sectors, sub-sectors, publics and myriad other groups such that comprehensive representation becomes practically impossible. The ‘Key Group Approach to Stakeholder Analysis’ presented here helps to address this issue by setting out a methodological approach, split over two rounds, that other researchers can use and adapt to their own contexts and research design needs. It has been based on the review of theoretical and conceptual developments across multiple sectors over the last 30 years, and it draws directly on the experience of operationalising a major six-year programme of research, which involved multiple iterations of stakeholder analysis and identification. The data from the programme’s evaluation workshops make clear the value of this approach, and together allow detailed reflection of the approach’s strengths and limitations. Not all elements are fully replicable in all contexts due to inevitable resource constraints compared to our 6-year programme, but all steps are relatively straight-forward and most gaps should be addressed through iterative application.

## Data Availability

The original contributions presented in the study are included in the article/supplementary material, further inquiries can be directed to the corresponding author/s.
