# Supplementary material for: Stakeholder analysis in urban-planetary health research: The Key Group Approach
Source: Front Public Health. 2025 Sep 24;13:1629249. doi: 10.3389/fpubh.2025.1629249 (PMC12506460; doi:10.3389/fpubh.2025.1629249)
Supplement: Supplementary file 1 [file Table_1.DOCX]

Supplementary Material

# Stakeholder categories for healthy urban development – final categories

| **REGION** | **PUBLIC SECTOR** | **PRIVATE SECTOR** | **THIRD** | **TECHNICAL DISCIPLINES** | PUBLIC | **NEW CATEGORIES** |
| --- | --- | --- | --- | --- | --- | --- |
| Wales | Cabinet Office | **Investors -** Pension funds / Insurance | Town planning | Urban planning | Geographic | Quality Deliverers |
| Scotland | Treasury | **Investors** - Real Estate | Transport | Transport | Statistical | Valuation Experts |
| West Midlands | **Dept -** Housing & Communities | **Investors** - Stock market | Housing | Property developer | Political | Radical Thinkers |
|  | **Dept** - Energy & Net Zero | **Investors** - Local Government | Health | Land/Real-estate | Experiential | Progressive Partners |
|  | **Dept** - Healthcare | **Investors** - Banks (including mortgage providers) | Economics | Housing |  | Land Experts |
|  | **Dept** - Transport | **Developers** - Commercial | Environment | Health |  | GBI Innovators |
|  | **Dept** - Ennvironment & Food | **Developers** - Residential | Construction | Finance/Investment |  | Policy Innovators |
|  | **Govt Body** - OHID | **Developers** - Independent | Community | Environment |  | Mediators |
|  | **Govt Body** - Homes England | Custom Build Housing | Policy (think tanks, advocacy, etc)? | Construction |  | Behaviour Change Experts |
|  | **Govt Body** - One Public Estate | **Land** - Land agents |  | Community |  | TRUUD Private Partners |
|  | **CA -** Spatial Planning | **Land** - Public Land (national & local) |  | Politics/ policymaking? |  | Transport Strategists |
|  | **CA** - Transport | **Land** - Private Land (Land owners) |  | Governance? |  | Progressive Planners |
|  | **CA** - Health | **Land** - Promoters |  |  |  | Systems Lawyers |
|  | **Local -** Planning | **Consultants** - Surveyors |  |  |  | Urban Futurists |
|  | **Local** - Property | **Consultants** - Accountants/ Management |  |  |  | Finance Innovators |
|  | **Local** - Major Projects | **Consultants** - Property Lawyers |  |  |  | Deliberative Democrats |
|  | **Local** - Health | **Consultants** - Transport |  |  |  |  |
|  | **Local** - Sustainability | **Consultants** - Planning/Design/Engineering |  |  |  |  |
|  |  | **Consultants** - Financial services |  |  |  |  |
|  |  | **Consultants** - Real estate brokers |  |  |  |  |

# Completed table used to develop Key Groups from themes, attributes and example roles

| **Do we have stakeholders who understand…** | |  |  |  |
| --- | --- | --- | --- | --- |
|  | **Specific attributes** | **Example roles** | **Shortened**  **Title** | **Description** |
|  |  |  |  |  |
| How to prioritise health in decision-making? | Clear understanding of how (and which specific aspects of) upstream urban development decision-making impacts on health outcomes.  Our evidence (from the pilot, certainly) suggested that most experienced urban practitioners are aware of what's healthy and what's not (e.g. high quality homes, low car, quality green space, etc.), but very few have a comprehensive understanding of what is needed to deliver high quality development - having both of those is relatively rare.  Those that have both, therefore, tend to be developers and/or developer-investors, or social impact/ESG leads working independently or with investors, delivering higher-end, premium quality 'products'. | **Private**: CEOs, Experienced Developers/Investors with Track Record in High Quality Developments, Directors Responsible for Social Impact and ESG, Experienced Consultants  **Public**: Senior officers from relevant departments at national and local level, Chief Planner, Treasury Lead responsible for Regen/Development, One Public Estate Lead  **Third**: Senior/experienced think tank policy specialists | **Quality Deliverers** | Many experienced urban practitioners are aware of what's healthy and what's not (e.g. high quality homes, low car, quality green space, etc.), but very few have a comprehensive understanding of what is needed to deliver high quality development - having both of those is relatively rare. Those that have both, therefore, tend to be developers and/or developer-investors, or social impact/ESG leads working independently or with investors, delivering higher-end, premium quality 'products', though even these tend to focus in high value locations where they can charge a premium. Most volume-builders have one or two high-end developments they can point to, and many have good aspects, but these are rare and often supported by the public purse. Delivering high quality in low value areas is very rare and usually small-scale and community-led (e.g. successful CLTs). |
| The need for economic valuation (and how to apply it)? | Familiarity with economic valuation and what it can (and can't) do. This requires a relatively nuanced understanding of economics or valuation approaches, including the use of assumptions and the uncertainties that engenders, and ideally environmental economics, given how much more uncertain that is. | As above, but...with appraisal experts (not just economists, but those aware of how to apply them in urban development) | **Valuation Experts** | Familiarity with economic valuation and what it can (and can't) do. This requires a relatively nuanced understanding of economics or valuation approaches, including the use of assumptions and the uncertainties that engenders, and ideally environmental economics, given how much more uncertain that is. |
| How to overcome short-term thinking? | Notoriously challenging: requires entirely new ways of governing so likely will need experience, systems thinking and innovation (and/or indigenous wisdom?) | As above, but...with innovative thinkers (e.g. RSA? Indigenous voice?) | **Cathedral Thinking Innovators** | Overcoming short-termism is a 'wicked problem': it requires entirely new ways of governing so likely will need experience, systems thinking and innovation. 'Cathedral thinking' is a concept that refers to those that built the cathedrals in the middle ages, which took 100s of years to complete, knowing they would never see the end result. |
| What balanced partnerships might look like? | Long experience of working across sectors with knowledge of what works and what doesn't in terms of high quality delivery (i.e. track record of delivering high quality working in partnership with opposite sector - can be public or private) | As above, though...more focus on public sector leaders (i.e. Major Projects, Transport Strategy, Economic Development) | **Progressive Partners** | Long experience of working across sectors with knowledge of what works and what doesn't in terms of high quality delivery (i.e. track record of delivering high quality working in partnership with opposite sector - can be public or private) |
| Issues of land control and value? | Detailed understanding of the land system: i.e. who controls it and what that means, how it is used and valued, the laws that govern its use, etc. | As above, but…with specialists in land control and valuation (e.g. chartered surveyors (RICS), property lawyers, third sector) | **Land Experts** | Detailed understanding of the land system: i.e. who controls it and what that means, how it is used and valued, the laws that govern its use, etc. |
| How to address the issue of maintaining the public realm? | Deceptively challenging: requires innovative approaches and solutions from 'outside the box' to build then maintain high quality green and blue infrastructure (GBI) (e.g. Milton Keynes GI Fund). It also requires those that may not be innovators, but are also key to the delivery - e.g. volume-house builders. | **Private**: Volume Housebuilders, Developers  **Public**: Cross-Sector group at national level (DEFRA, MHCLG, DoH)? Local Parks/GI Leads, Biodiversity Net Gain Experts, Public Health, Nature Partnerships,  **Third**: RSA-type thinkers, Biodiversity by Design, Wildlife Trusts, etc. | **GBI Innovators** | Deceptively challenging: requires innovative approaches and solutions from 'outside the box' to build then maintain high quality green and blue infrastructure (GBI) (e.g. Milton Keynes GI Fund). It also requires those that may not be innovators, but are also key to the delivery - e.g. volume-house builders. |
| The role of government in this challenge space? | Experienced in Government generally, and familiar with cross-departmental working and policy innovation. Would tend to be highly talented mid-career government officers or policy think tank consultants, or those who are very experienced yet still passionate about change. As with the Quality Deliverers, this is quite rare. | As above, plus…policy innovators | **Policy Innovators** | Experienced in Government generally, and familiar with cross-departmental working and policy innovation. Would tend to be highly talented mid-career government officers or policy think tank consultants, or those who are very experienced yet still passionate about change. As with the Quality Deliverers, this is quite rare. |
| Corporate behaviour change | Need understanding of both management/decision-making and psychology, but also ideally a good understanding of the context of urban development in terms of how it might be reasonably applied (e.g. how investors, developers and consultants think and make decisions, and why). | Usually academics or consultant psychologists, ideally with clear expertise in practical application (e.g. NESTA/BIT), but also a clear understanding of its limitations. | **Behaviour Change Experts** | Need understanding of both management/decision-making and psychology, but also ideally a good understanding of the context of urban development in terms of how it might be reasonably applied (e.g. how investors, developers and consultants think and make decisions, and why). |
| City-region transport planning | Similar to the Quality Deliverers, but for transport, these people need to be both well aware of the links between health and transport (e.g. air pollution, noise, sedentary lifestyles, etc.), which is relatively well known about now, as well as - and this is rarer - highly experienced in terms of strategic and major transport infrastructure provision, including the significant challenges (e.g. financial, political, spatial). It would typically be very experienced academics, transport planners, engineers and investors. | **National**: DfT, Highways Agency, Transport Scotland, Scottish Futures Trust, Transport Wales, Infrastructure Engineering  **Private - Companies**: First, GWR, etc.  **Private - Consultants:** Chartered Institute of Highways and Transportation (CIHT), Transport Planning Society (TPS), Chartered Institute of Logistics and Transport (CILT)  **Combined Authority**: TfL, TfGM, WMCA Transport, WECA Transport  **Local**: ADEPT, Cardiff, Edinburgh, Bristol  **Third**: Sustrans, CfBT  **Academic**: DfT College of Experts | **Transport Strategists** | Similar to the Quality Deliverers, but for transport, these people need to be both well aware of the links between health and transport (e.g. air pollution, noise, sedentary lifestyles, etc.), which is relatively well known about now, as well as - and this is rarer - highly experienced in terms of strategic and major transport infrastructure provision, including the significant challenges (e.g. financial, political, spatial). It would typically be very experienced academics, transport planners, engineers and investors. |
| Spatial planning for large-scale development | There are many planners, urban designers, architects and other technical professionals that are familiar with spatial planning, and most should be aware of the challenges and latest guidance. They are important knowledge holders, but there is a tendency amongst this group to assume - given how core this is to urban development generally - that plans and planning policy on their own will solve all the issues, so these should not dominate discussions. | **National**: MHCLG  **Private**: Consultants, especially large engineering firms, but also large planning and architectural practices  **Combined Authority**: GMCA, WMCA, WECA  **Local**: Cardiff, Edinburgh, Birmingham  **Third**: TCPA, UDG, RTPI, RIBA  **Academic**: Spatial planners | **Progressive Planners** | There are many planners, urban designers, architects and other technical professionals that are familiar with spatial planning, and most should be aware of the challenges and latest guidance. They are important knowledge holders, but there is a tendency amongst this group to assume - given how core this is to urban development generally - that plans and planning policy on their own will solve all the issues, so though essential, they should not dominate discussions. |
| Law and planning | The obvious areas of the law relate to planning or housing, however, there are broader categorisations of the law - social welfare, environment - that span multiple areas of law, which are highly relevant, if not more so (e.g. corporate law, constitutional law). Senior practicing lawyers may have a very clear understanding of their area of the law and its area of application, but not a clear understanding of the broader systems at play and the innovation needed. |  | **Systems Lawyers** | The obvious areas of the law relate to planning or housing, however, there are broader categorisations of the law - social welfare, environment - that span multiple areas of law, which are highly relevant, if not more so (e.g. corporate law, constitutional law). Senior practicing lawyers may have a very clear understanding of their area of the law and its area of application, but not a clear understanding of the broader systems at play and the innovation needed. |
| The role of (fast growing) cities | Cities, and especially fast growing cities, are where most development is taking place in the UK: demand is high and there is considerable money available for investment, especially FDI (foreign direct investment) from China and the Middle East. There is also currently considerable pressure for: a) levelling up, given inequality and clear dominance of London and the South East, and b) devolution, given how centralised the UK is, and the success in recent years of some metro mayors, notably Manchester and Birmingham. However, smaller cities should not be forgotten, and rural living still makes up a substantial minority of the UK population, with considerable pressure too on greenfield development from the volume housebuilders especially, but also via the Government in terms of their New Towns agenda. As such, we need city champions, but also those who understand the importance of and challenges facing smaller cities in terms of transport, planning, densification and green infrastructure. | As with Progressive Planners above, but also...Centre for Cities, Core Cities | **Urban-Rural Futurists** | Cities, and especially fast growing cities, are where most development is taking place in the UK: demand is high and there is considerable money available for investment, especially FDI (foreign direct investment) from China and the Middle East. There is also currently considerable pressure for: a) levelling up, given inequality and clear dominance of London and the South East, and b) devolution, given how centralised the UK is, and the success in recent years of some metro mayors, notably Manchester and Birmingham. However, smaller cities should not be forgotten, and rural living still makes up a substantial minority of the UK population, with considerable pressure too on greenfield development from the volume housebuilders especially, but also via the Government in terms of their New Towns agenda. Many people want to live in nature, but have access to the city. As such, we need city champions, but also those who understand the importance of and challenges facing smaller cities and towns in terms of transport, planning, densification and green infrastructure/biodiversity, as well as realism about what's achievable: i.e. walkable concrete communities vs WFH alone in the countryside? |
| Investment (in relation to development) | This is partially covered above, but needs more substantive input/coverage as it's a vast sector in its own right so shouldn't be bundled in to development with Quality Deliverers even though there is a strong overlap (e.g. pension funds with in-house property sections, real estate investors, real estate investment trusts (REITs)). There are many other financial operators (e.g. banks, insurance providers, mortgage providers, financial think tanks and innovators) who are essential to bring in, though it's not easy given most are unfamiliar with the nuances of urban development. |  | **Finance Innovators** | This is partially covered above, but needs more substantive input/coverage as it's a vast sector in its own right so shouldn't be bundled in to development with Quality Deliverers even though there is a strong overlap (e.g. pension funds with in-house property sections, real estate investors, real estate investment trusts (REITs)). There are many other financial operators (e.g. banks, insurance providers, mortgage providers, financial think tanks and innovators) who are essential to bring in, though it's not easy given most are unfamiliar with the nuances of urban development. |

# Final, simplified template shared internally and externally

| **SECTOR** | **TIER** | **AREA** | **First Name** | **Surname** | **Position** | **Organisation** | **KEY GROUPS*** |
| --- | --- | --- | --- | --- | --- | --- | --- |
| **PUBLIC** | **UK-WIDE** | Government  Finance  Financial oversight |  |  |  |  |  |
|  | **NATIONAL** | Housing & Communities  Energy & Net Zero  Healthcare  Transport  Environment & Food  Land |  |  |  |  |  |
|  | **COMBINED AUTHORITIES**  **(ENGLAND ONLY)** | Spatial Planning  Transport  Health |  |  |  |  |  |
|  | **LOCAL** | Planning  Property  Major Projects  Health  Sustainability |  |  |  |  |  |
| **PRIVATE** | **INVESTORS** | Pension funds / Insurance  Real Estate Investors  Banks |  |  |  |  |  |
|  | **DEVELOPERS** | Commercial  Residential  Independent  Custom Build |  |  |  |  |  |
|  | **LAND** | Agents  Promoters |  |  |  |  |  |
|  | **CONSULTANTS** | Surveyors  Accountants / Management  Property Lawyers  Transport  Planning / Design / Engineering   Real estate brokers |  |  |  |  |  |
| **THIRD** | **CHARITIES, THINK TANKS, ETC.** | Town planning  Transport  Energy & Net Zero  Housing  Health  Economics  Environment  Construction  Community   Policy |  |  |  |  |  |
|  | **ACADEMIC** |  |  |  |  |  |  |
| **PUBLIC** |  | Representative:  • Geographically  • Statistically  • Politically  • Experientially |  |  |  |  |  |
